# KLF13 Regulates the Activity of the GH-Induced JAK/STAT Signaling by Targeting Genes Involved in the Pathway

**DOI:** 10.3390/ijms241311187

**Published:** 2023-07-07

**Authors:** José Ávila-Mendoza, Karen Delgado-Rueda, Valeria A. Urban-Sosa, Martha Carranza, Maricela Luna, Carlos G. Martínez-Moreno, Carlos Arámburo

**Affiliations:** Departamento de Neurobiología Celular y Molecular, Instituto de Neurobiología, Campus Juriquilla, Universidad Nacional Autónoma de México, Querétaro 76230, Mexico

**Keywords:** Krüppel-like factors, JAK/STAT, growth hormone, hippocampal neurons, signal transduction

## Abstract

The Krüppel-like factor 13 (KLF13) has emerged as an important transcription factor involved in essential processes of the central nervous system (CNS). It predominantly functions as a transcriptional repressor, impacting the activity of several signaling pathways with essential roles in the CNS, including the JAK/STAT pathway, which is the canonical mediator of growth hormone (GH) signaling. It is now recognized that GH has important actions as a neurotrophic factor. Therefore, we analyzed the effects of KLF13 on the activity of the JAK/STAT signaling pathway in the hippocampus-derived cell line HT22. Results showed that KLF13 directly regulates the expression of several genes involved in the JAK-STAT pathway, including *Jak1*, *Jak2*, Jak3, and *Socs1*, by associating with their proximal gene promoters. In addition, it was found that in KLF13-deficient HT22 neurons, the expression of *Jak1*, *Stat3*, *Socs1*, *Socs3*, and *Igf1* was dysregulated, exhibiting mRNA levels that went up to 7-fold higher than the control cell line. KLF13 displayed a differential effect on the GH-induced JAK/STAT pathway activity, decreasing the STAT3 branch while enhancing the STAT5 branch. In KLF13-deficient HT22 cells, the activity of the STAT3 branch was enhanced, mediating the GH-dependent augmented expression of the JAK/STAT output genes *Socs1*, *Socs3*, *Igf1*, and *Bdnf*. Furthermore, GH treatment increased both the nuclear content of KLF13 and *Klf13* mRNA levels, suggesting that KLF13 could be part of the mechanisms that maintain the homeostatic state of this pathway. These findings support the notion that KLF13 is a regulator of JAK/STAT activity.

## 1. Introduction

The Krüppel-like factors (KLFs) constitute a family of eighteen transcription factors characterized by three C-terminal C2H2 zinc finger motifs that recognize GC/GT-rich sequences in DNA [1]. They are grouped into three subfamilies based on the primary structure of their N-terminal domains, which are highly variable and comprise sites for interaction with coregulators that modulate, in part, the activities of KLFs [2]. KLFs have emerged as transcription factors with important functions in nervous system physiology, including the proliferation of neuronal precursors, their differentiation, axon elongation, and regeneration [3,4,5]. This work focused on KLF9 and KLF13, members of subfamily 3, which share a common domain that interacts with the co-repressor protein Swi-independent 3a (Sin3a) [6]. We recently discovered that expression of *Klf13* increases in the hippocampus during the postnatal development of mice, in a way similar to its paralogous gene *Klf9*, which is in accordance with their ability to promote and maintain neuronal differentiation [7,8,9].

To understand the mechanisms underlying the actions of these factors on neurons, previous studies have described that, like KLF9, KLF13 functions predominantly as a transcriptional repressor by associating with chromatin within proximal promoters of its target genes [10,11]. However, they can also induce gene transcription depending on the chromatin state and cellular context [2,11,12]. Furthermore, genome-wide analyses have revealed that KLF13 regulates approximately four times more genes than KLF9 and that it directly represses the transcription of genes involved in neurotrophic factor signaling pathways (e.g., PKA-cAMP, PI3K-AKT, and JAK-STAT) in mouse hippocampus-derived neurons [8,10,11]. We recently confirmed that KLF13 globally reduces the induced activity of the cAMP signaling pathway by repressing the expression of several genes associated with this pathway [8]. However, no validation of KLF13’s effects on other pathways has been performed.

The JAK-STAT signaling pathway plays a crucial role in cell growth, survival, development, and differentiation [13]. Deregulation of this pathway has been associated with various pathologies, including cancers, immune disorders, and cardiovascular diseases [14]. In the CNS, the JAK-STAT signaling pathway is primarily associated with gene regulation during development, while in adulthood, it has been involved in processes such as neuroinflammation, neurogenesis, and neuroregeneration [15]. This pathway is activated by several polypeptides, including hormones, growth factors, or cytokines [16]. It is known that growth hormone (GH) activates the JAK/STAT signaling pathway [17]. GH signaling is induced by binding to its specific membrane receptor (GHR) followed by the activation of Janus Kinase (JAK), which then autophosphorylates and transphosphorylates the complementary GHR-JAK2 complex [18,19]. This leads to the recruitment of transcription factors from the Signal Transducer and Activator of Transcription (STATs) family, which are phosphorylated, dimerized, and translocated to the nucleus to regulate the expression of target genes [20]. Some of the early-activated genes include members of the Suppressor of Cytokine Signaling (*Socs*) family genes, whose protein products exert negative feedback to regulate the JAK/STAT activity [21].

GH is well known for its primary actions in regulating postnatal somatic growth [22]. In addition, accumulating evidence suggests that GH plays essential roles in CNS development [23,24] and has neurotrophic effects in adults [25,26] by activating the JAK/STAT signaling pathway. These actions include neuroprotection in injury models of hypoxia-ischemia [27,28,29], excitotoxicity [30], trauma [25,31], or stroke [32]. These effects can be exerted directly by GH but can also be regulated by other neurotrophic factors that increase their expression in response to GH. The most recognized mediator of GH actions is Insulin-like Growth Factor Type 1 (IGF1) [26,33], although it is now known that GH can also promote the expression of other neurotrophins, such as the Brain-Derived Neurotrophic Factor (BDNF) and Neurotrophin-3 (NT-3) [30], suggesting that they may contribute to GH actions on neurons.

To gain insight into the molecular mechanisms underlying KLF13 regulation on the JAK/STAT signaling pathway, we analyzed the effects of overexpressing or depleting KLF13 in the HT22 cell line derived from the adult mouse hippocampus upon the GH-induced activity of this pathway. The results of this study provide valuable insights into the regulatory mechanisms of the JAK/STAT pathway and present an exciting possibility for enhancing its activity to optimize its beneficial effects.

## 2. Results

### 2.1. KLF13 Regulates Several Genes Involved in the JAK/STAT Pathway in HT22 Cells

**Figure 1:**
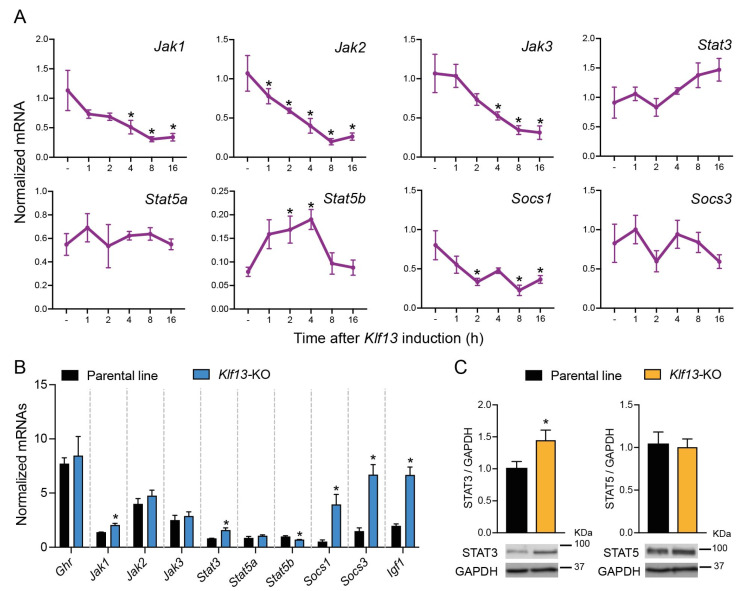
KLF3 regulates the expression of genes involved in the JAK/STAT signaling pathway. (**A**). The HT22-TR/TO-*Klf13*-cell line was treated with vehicle or doxycycline (dox; 1 μg/mL) to force the expression of *Klf13* transgene. We harvested cells at indicated times after dox treatment, isolated RNA, and conducted RT-qPCR. Forced induction of *KLF13* caused time-dependent gene repression of *Jak1*, *Jak2*, *Jak3*, and *Socs1*, while *Stat5b* expression was induced 2 h after dox treatment, peaked at 4 h, and then returned to baseline after 8 h of *Klf13* induction (*Jak1*: *F*_(5,17)_ = 6.967, *p* = 0.001; *Jak2*: *F*_(5,17)_ = 13.50, *p* < 0.0001; *Jak3*: *F*_(5,17)_ = 10.03, *p* = 0.0001; *Socs1*: *F*_(5,17)_ = 5.106, *p* = 0.0049; and *Stat5b*: *F*_(5,18)_ = 4.588, *p* = 0.0071; one way ANOVA. Asterisks indicate statistically significant differences with *p* < 0.05 in Dunnett’s post hoc test comparing the mean of treatments with the control). Points represent the mean ± SEM (*n* = 4/time point). There was no statistically significant difference in the expression *of Stat3, Stat5a*, and *Socs3* upon treatment with dox. (**B**). The mRNA levels of *Jak1*, *Stat3*, *Socs1*, *Socs3*, and *Igf1* were increased, while *Stat5b* decreased in HT22-*Klf13*-KO cells compared with the parent cell line (*Jak1*, *p* = 0.0055; *Stat3*, *p* = 0.0052; *Stat5a*, *p* = 0.0167; *Socs1*, *p* = 0.0116; *Socs3*, *p* = 0.0006; and *Igf1*, *p* = 0.0002; Student’s *t*-test. Asterisks indicate statistically significant differences with *p* < 0.05). Bars represent the mean ± SEM (*n* = 4/genotype). The mRNAs of *Ghr*, *Jak2*, *Jak3*, and *Stat5b* were unaffected by the loss of *Klf13*. (**C**). The STAT3 protein content was higher in HT22-*Klf13*-KO cells compared with the parental line, while STAT5 protein levels remained unchanged (STAT3, *p* = 0.048; Student’s *t*-test. Asterisks indicate statistically significant differences with *p* < 0.05). Bars represent the mean ± SEM (*n* = 4/genotype).

Our previous publication on RNA-seq transcriptome analysis in HT22 cells [10] revealed that the forced expression of KLF13 impacts several signaling pathways, including JAK/STAT. In this study, we used our previously engineered cell line to force the expression of KLF13 with doxycycline and validated the effects of KLF13 on the expression of JAK/STAT genes during a time-course experiment. Through an RT-qPCR targeted analysis, we observed a time-dependent decrease in the mRNA levels of *Jak1*, *Jak2*, *Jak3*, and *Socs1*, which were up to 73 ± 10.1, 81.5 ± 3.6, 70.7 ± 7.4, and 72 ± 8% lower, respectively, in comparison with their corresponding controls, between 8 and 16 h after *Klf13* induction (Figure 1A). Conversely, the expression *Stat5b* was induced by KLF13, as evidenced by an increase in its mRNA levels peaking at 2 h post doxycycline treatment, maintained at 4 h (2.4-fold), and returning to basal levels 8 h after *Klf13* induction (Figure 1A). Results also showed that other genes involved in the JAK/STAT signaling pathway, such as *Stat3*, *Stat5a*, and *Socs3*, were unaffected by the forced expression of *Klf13* (Figure 1A).

### 2.2. The Expression of Several Genes Involved in the JAK/STAT Pathway Is Dysregulated in KLF13-Deficient HT22 Cells

The previously engineered HT22-*Klf13*-KO cell line, in which the *Klf13* gene was mutated using CRISPR-Cas9 [10], was used to assess the impact of KLF13 depletion on JAK/STAT gene expression. Results showed that, among the genes exhibiting differential expression after forced expression of *Klf13*, only *Jak1*, *Stat5b*, and *Socs1* displayed dysregulated expression in the absence of KLF13 (Figure 1B). The mRNA levels of *Jak1* and *Socs1* were 1.4 ± 0.12-fold and 7.8 ± 0.12-fold higher, respectively, in *KLf13*-KO cells compared to the parental line, while *Stat5b* showed a 30.54 ± 3.5% decrease in KLF13-depleted cells. In addition, we observed that mRNA levels of *Stat3*, *Socs3*, and *Igf1*, which showed no changes after forced expression of *Klf13*, were upregulated by 1.9 ± 0.13 (*Stat3*), 4.4 ± 0.13 (*Socs3*), and 3.4 ± 0.1 (*Igf1*)- fold, respectively, in *Klf1*3-KO HT22 cells (Figure 1B). On the other hand, the mRNA levels of *Ghr*, *Jak2*, *Jak3*, and *Stat5a* showed no changes due to KLF13 depletion (Figure 1B).

In addition to the RT-qPCR targeted analysis, we also assessed the effect of KLF13 depletion on the protein levels of STAT3 and STAT5 in HT22 cells, which are effectors of the JAK/STAT signaling pathway. Results demonstrated that the protein extracts of *Klf13*-KO cells exhibited a significant increase (1.4 ± 0.1-fold) in STAT3 compared to the parental line, while levels of STAT5 showed no differences between the two cell lines (Figure 1C).

### 2.3. KLF13 Associates in the Chromatin with Promoters of Jak1, Stat5b, Socs1, and Socs3 Genes in HT22 Cells

**Figure 2:**
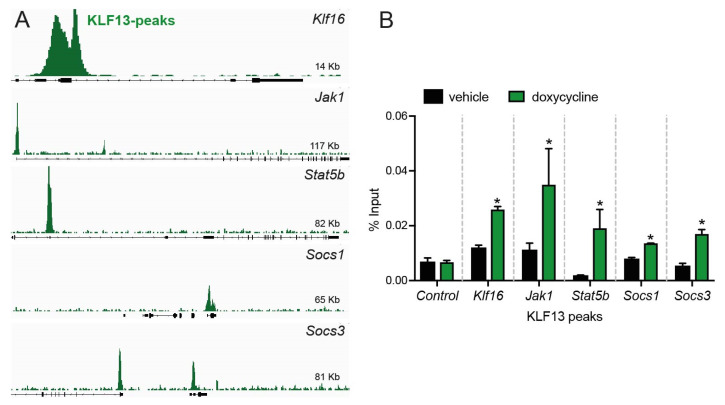
KLF13 associates in chromatin with promoters of some genes involved in the JAK/STAT signaling pathway in HT22 cells. (**A**). Genome traces from the Integrative Genome Viewer (IGV) were captured as screenshots to show the precise locations of KLF13 chromatin-streptavidin precipitation sequencing (ChSP-seq) peaks. The peaks were identified at genes in the JAK/STAT signaling pathway [10]. The gene structures are presented below in the genome traces, where black-filled bars and lines represent exons and introns, respectively. All peaks are located within the proximal promoter regions of the genes, and the gene orientations are 5’ to 3’. (**B**). To confirm the KLF13 ChSP-seq peaks associated with JAK/STAT signaling pathway genes, we conducted targeted ChIP-qPCR assays in HT22 cells. HT22-TR/TO-*Klf13* cells were treated with either vehicle or doxycycline (dox; 1 μg/mL) for 16 h. After harvesting the cells, chromatin was isolated for ChIP assays. We used the Klf16 intron, which did not have KLF13 ChSP peaks, as a negative control region (Control). The KLF13 ChIP-qPCR data are expressed as a percentage of the input. All the *loci* examined showed enrichment after forced expression of *Klf13* in the KLF13 ChIP-qPCR assay (*Klf16*, *p* = 0.0005; *Jak1*, *p* = 0.0394; *Stat5b*, *p* = 0.0258; *Socs1*, *p* = 0.0022; and *Socs3*, *p* = 0.0021; Student’s *t*-test. Asterisks indicate statistically significant differences with *p* < 0.05). Bars represent the mean + SEM (*n* = 4/genotype).

Previously, ChSP-seq was used to analyze the genome-wide association of KLF13 with chromatin in HT22 cells [10]. The dataset from that study was re-analyzed in this study, and results revealed that KLF13 was associated with genomic regions within 1 kb of the transcription start sites of *Klf16* (control), *Jak1*, *Stat5b*, *Socs1*, and *Socs3* (Figure 2A). To further investigate the association of KLF13 with these genes, we performed targeted ChIP-qPCR assays for KLF13 on chromatin isolated from HT22-TR/TO-*Klf13* cells treated with doxycycline for 16 h. The results revealed robust increases in KLF13 ChIP signals (2.8 ± 0.12-fold) at the *Klf16* promoter (positive control) but no change at the *Klf16* intron (negative control). Furthermore, we observed statistically significant increases in the KLF13 ChIP signal at the promoters of each of the four genes tested (Figure 2B): *Jak1* (3.4 ± 0.2-fold), *Stat5b* (12 ± 0.3-fold), *Socs1* (1.8 ± 0.01-fold), and *Socs3* (3.2 ± 0.06-fold).

### 2.4. KLF13 Differentially Regulates GH-Dependent Activity of the STAT3 and STAT5 Branches in the JAK/STAT Pathway

**Figure 3:**
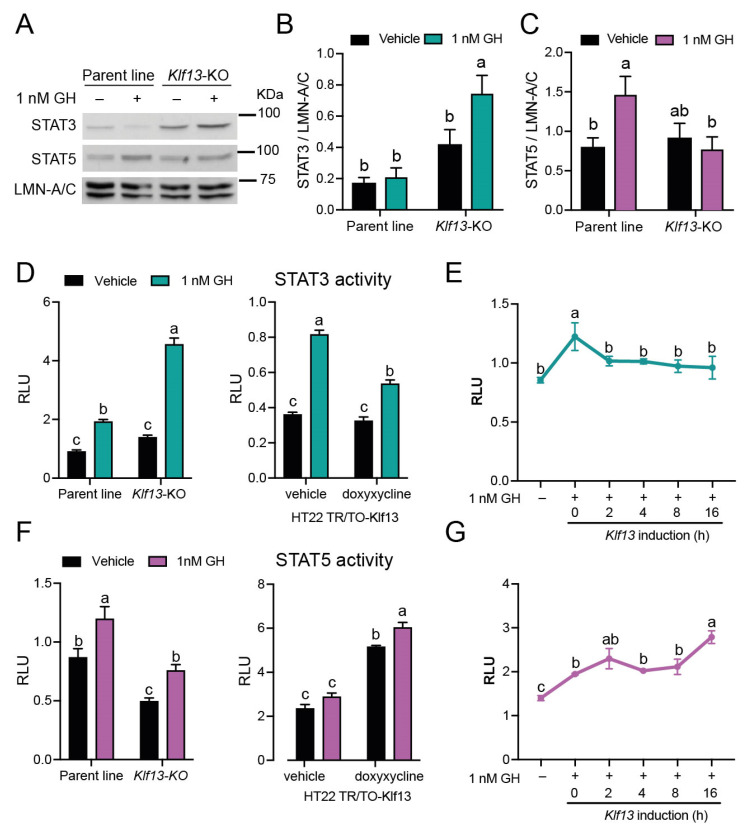
KLF13 differentially regulates GH-dependent JAK/STAT activity by repressing the STAT3 branch and inducing the STAT5 branch in HT22 cells. (**A**). We evaluated the JAK/STAT activity by analyzing the nuclear translocation of STAT3 and STAT5 induced by 1 nM GH in both parental and *Klf13*-KO HT22 cell lines. After one hour of treatment, cells were harvested, and nuclear fractions were extracted and resolved using SDS/PAGE and Western blot; STAT3 and STAT5 were analyzed in the nucleus using laminin A/C (LMN-A/C) as a loading control. (**B**). Quantification of STAT3 signal from panel (**A**). Basal STAT3 levels in the nucleus appear to be increased in *KLF13*-KO cells, although not significantly different. GH treatment strongly induces the STAT3 translocation in *Klf13*-KO cell line, while in the parental line (PL), GH had no effect on STAT3 translocation (PL-Ve vs. *Klf13*-KO-GH, *p* = 0.0005; PL-GH vs. *Klf13*-KO-GH, *p* = 0.0012; and *Klf13*-Ve vs. *Klf13*-KO-GH *p* = 0.0235; two-way ANOVA followed by Tukey’s multiple comparison post hoc test). (**C**). Quantification of STAT5 signal from panel (**A**). Basal levels of STAT5 are unaffected by KLF13 depletion, and GH treatment induced STAT5 translocation only in parental but not in Klf13-KO cell lines (PL-Ve vs. PL-GH, *p* = 0.0259; and PL-GH vs. *Klf13*-KO-GH, *p* = 0.0219; two-way ANOVA followed by Tukey’s multiple comparison post hoc test). Bars represent the mean ± SEM (*n* = 4/genotype), and treatments with different letters (a, b or c) indicate statistically significant differences (*p* < 0.05). Means with the same letter are not significantly different. (**D**–**G**). We performed transfection reporter assays to validate the effects of KLF13 deficiency or forced expression on JAK/STAT activity. The HT22 parent line, *Klf13-*KO, and TR/TO-*Klf13* were co-transfected with pRenilla plus the firefly luciferase reporter vectors pGL-GAS and pGL-STAT5, which serve as sensors for STAT3 and STAT5 activity, respectively. The relative luciferase activity (RLA) represents firefly luciferase normalized to the *Renilla* luciferase values. (**D**). Left, GH-dependent STAT3 activity is strongly enhanced in *Klf13*-KO cells compared to parent line (PL-Ve vs. PL-GH, *p* = 0.0004; PL-Ve vs. *Klf13*-KO-GH, *p* < 0.0001; PL-GH vs. *Klf13*-KO-Ve, *p* = 0.0438; PL-GH vs. *Klf13*-KO-GH, *p* < 0.0001; and *Klf13*-Ve vs. *Klf13*-KO-GH, *p* < 0.0001). Right, the STAT3 activity induced by GH is reduced by simultaneous induction of KLF13 with doxycycline (Ve-Ve vs. Ve-GH, *p* < 0.0001; Ve-Ve vs. Dox-GH, *p* = 0.0004; Ve-GH vs. dox-Ve, *p* < 0.0001; Ve-GH vs. dox-GH, *p* < 0.0001; dox-Ve vs. dox-GH, *p* < 0.000). (**E**). The STAT3 activity induced by 20 h of GH treatment is blocked when Klf13 expression is forced from 2 to 16 h prior to harvesting cells (*F*_(5,18)_ = 3.124, *p* = 0.033). (**F**). Left, the basal STAT5 activity is lower in *Klf13*-KO cells compared to the parent line, and although GH induces STAT5 activity in both cell genotypes, the absolute activity is lower in the absence of KLF13 (PL-Ve vs. PL-GH, *p* = 0.0493; PL-Ve vs. *Klf13*-KO-Ve, *p* = 0.0012; PL-GH vs. *Klf13*-KO-Ve, *p* < 0.0001; PL-GH vs. *Klf13*-KO-GH, *p* < 0.0059; and *Klf13*-KO-Ve vs. *Klf13*-KO-GH, *p* < 0.0095). Right, the forced expression of KLF13 induces the STAT5 activity, which is further increased by concurrent GH treatment (Ve-Ve vs. dox-Ve, *p* < 0.0001; Ve-Ve vs. dox-GH, *p* < 0.0001; Ve-GH vs. dox-Ve, *p* < 0.0001; Ve-GH vs. dox-GH, *p* < 0.0001; and dox-GH vs. dox-GH, *p* = 0.0199). (**G**). The STAT5 activity induced by 20 h of GH treatment is enhanced when *Klf13* expression is induced by 2 and 16 h prior to harvesting cells (*F*_(5,18)_ = 14.58, *p* < 0.0001). We performed two-way ANOVA followed by Tukey’s multiple comparison post hoc test for panels (**D**,**E**), while one-way ANOVA followed Dunnett’s post hoc test was conducted for panels (**E**,**G**).

After observing that KLF13 has a regulatory role in the expression of multiple JAK/STAT genes, including those that directly regulate through associating with target gene promoters, the impact of KLF13 on the activity of the signaling pathway in both the STAT3 and STAT5 branches was studied. We first analyzed the nuclear protein content of STAT3 and STAT5 by Western blotting after 1 h of GH treatment in both the parental and *Klf13*-KO cell lines. Results showed that the baseline level of STAT3 was apparently higher in *Klf13*-KO cells compared with the parental line, as shown in Figure 3A, although this difference was not statistically significant (*p* = 0.0681, Figure 3B). For STAT3, two-way ANOVA revealed statistically significant main effects of genotype (*F*_(1,12)_ = 120.16, *p* < 0.0007), and multiple comparison analyses showed that GH treatment induced an increase of 1.7 ± 0.1-fold in the STAT3 signal in *Klf13*-KO cells compared with vehicle-treated cells, but no effect was seen in the parental cell line (Figure 3B).

The basal levels of STAT5 in the nucleus were unaffected by the *Klf13* mutation (Figure 3A,C). Furthermore, the two-way ANOVA showed no significant effects of genotype or GH treatment. However, individual comparison in Dunnet’s post hoc analysis revealed that GH treatment increased the protein content of STAT5 by 2 ± 0.07-fold in the nucleus of the parental line but not in *Klf13*-KO cells (Figure 3C).

To further investigate the role of KLF3 in the activity of the JAK/STAT pathway, we conducted transfection reporter assays using the plasmid vectors pGL-GAS and pGL-STAT5. These plasmids were previously validated as sensors of the activity of STAT3 and STAT5, respectively [34,35]. Cells of the different genotypes: parental, *Klf13*-KO, or TRT/TO-*Klf13* were transfected, either with plasmids pGL-GAS or pGL-STAT5, to assess the GH-induced activity of STAT3 and STAT5 in the presence of different levels of KLF13. By using two-way ANOVA, we found significant effects of genotype (*F*_(1,12)_ = 73.90, *p* < 0.0001), GH treatment (*F*_(1,12)_ = 156.5, *p* < 0.0001), and interaction between them (*F*_(1,12)_ = 282.8, *p* < 0.0001) in the parental line as compared to *Klf13*-KO. This analysis also revealed that, although GH induced the STAT3 activity in the parent line (2.1 ± 0.03-fold), the KLF13 depletion strongly enhanced the GH-dependent activity of STAT3 up to 3.4 ± 0.04-fold in HT22 cells (Figure 3D, left). Consistent with these results, our findings in TR/TO-*Klf13* cells showed that co-treatment of GH and doxycycline (*Klf13* induction) reduced the GH-dependent STAT3 activity from 2.6 ± 0.02- to 1.6 ± 0.04-fold in vehicle and doxycycline-treated cells, respectively. Despite the forced expression of *Klf13* reduced the STAT3 activity, the strong effects of genotype (*F*_(1,12)_ = 33.62, *p* < 0.0001), GH treatment (*F*_(1,12)_ = 55.98, *p* < 0.0001), and their interaction (*F*_(1,12)_ = 248.5, *p* < 0.0001) were still observed in this cell line (Figure 3D, right). To investigate whether KLF13 could inhibit STAT3 activity following GH induction, we stimulated *Klf13* expression (via doxycycline treatment) in HT22-TR/TO-*Klf13* cells at different time points after GH treatment. Results showed that the STAT3 activity induced after 20 h of GH treatment (1.4 ± 0.08-fold) was effectively blocked when cells were treated with doxycycline for 16 h. This means that after 4 h of GH treatment, doxycycline was added, and the cells were cultured for an additional 16 h. Notably, this inhibitory effect on STAT3 activity was sustained even when *Klf13* was induced for just 2 h (18 h GH treatment, followed by doxycycline treatment and 2 more hours of culturing). These findings indicate that KLF13 is capable of blocking STAT3 activity after its induction (Figure 3E).

On the other hand, we found that the basal activity of STAT5, as measured by the relative luciferase activity in cells transfected with the pGL-STAT5 plasmid vector, was reduced to 0.56 ± 0.05-fold in *Klf13*-KO cells compared to the parental line. Although GH induced STAT5 activity in both cell lines, the absolute activity was lower by 63.3 ± 6.7% in the absence of KLF13 (Figure 3F, left). The two-way ANOVA results showed significant main effects of genotype (*F*_(1,12)_ = 43.48, *p* < 0.0001) and GH treatment (*F*_(1,12)_ = 23.76, *p* = 0.0004) but not significant interaction effect. In line with these findings, forced expression of KLF13 alone led to a 2.2 ± 0.01-fold increase in the basal activity of STAT5. Moreover, this effect was further amplified by co-treatment with GH, resulting in a 2.6 ± 0.013-fold increase in activity compared to vehicle-treated cells (Figure 3F, right). In this cell line, we also observed significant main effects of genotype (*F*_(1,12)_ = 283.1, *p* < 0.0001) and GH treatment (*F*_(1,12)_ = 15.95, *p* = 0.0004) but not significant interaction effect. Finally, the experiment of inducing *Klf13* expression after several GH treatment time points demonstrated that the 1.4 ± 0.005-fold activity of STAT5 induced by GH was enhanced in a time-dependent manner, with activity increasing up to 1.9 ± 0.03-fold when cells were treated with doxycycline for 16 h before harvesting (Figure 3G).

### 2.5. The GH-Induced Expression of the JAK/STAT Output Genes Socs1, Socs3, Igf1, and Bdnf Is Enhanced in KLF13-Deficient HT22

**Figure 4:**
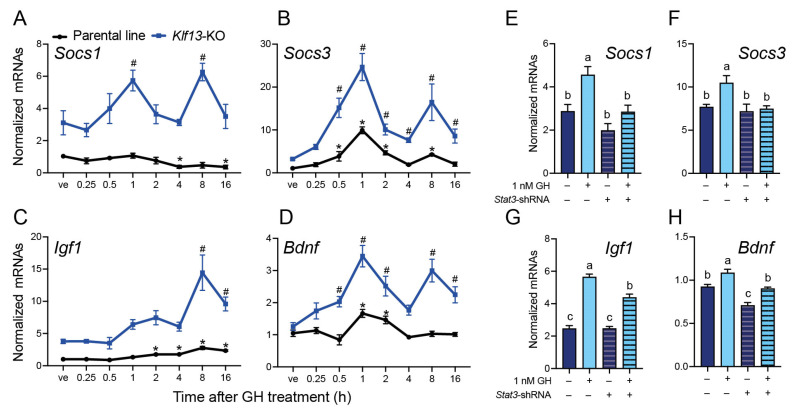
The GH-induced expression of JAK/STAT output genes is enhanced in KLF13 deficient HT22 cells, and this effect is mediated by STAT3. (**A**–**D**). A GH time-response experiment in *Klf13*-KO HT22 cells was performed, using the parental line as control. Cells of both genotypes were treated with 1 nM GH, harvested at indicated times, and subjected to RNA isolation and RT-qPCR to quantify the expression of the JAK/STAT output genes *Socs1* (**A**), *Socs3* (**B**), *Igf1* (**C**), and *Bdnf* (**D**). In the parent line (black lines), GH treatment caused a decrease in the mRNA levels of *Socs1* at 4 h and 16 h post-treatment, while the mRNAs of *Socs3*, *Igf1*, and *Bdnf* were increased at different time points after GH treatment (*Socs1*: *F*_(7,24)_ = 4.065, *p* = 0.0045; *Socs3*: *F*_(7,24)_ = 24.57, *p* < 0.0001; *Igf1*: *F*_(7,24)_ = 33.20, *p* < 0.0001; and *Bdnf*: *F*_(7,24)_ = 7.296, *p* = 0.0001). Treatment with GH in the HT22*-Klf13*-KO cell line (blue lines) also caused changes in the mRNA levels of the JAK/STAT output genes (*Socs1*: *F*_(7,24)_ = 4.167, *p* = 0.0039; *Socs3*: *F*_(7,24)_ = 10.03, *p* < 0.0001; *Igf1*: *F*_(7,24)_ = 9.237, *p* < 0.0001; and *Bdnf*: *F*_(7,24)_ = 7.877, *p* = 0.0001). The effect of GH on the expression of JAK/STAT output genes was strongly enhanced in the HT22*-Klf13*-KO cell line compared to the parental line. Statistical analysis was performed using one-way ANOVA followed by Dunnett’s post hoc test. Asterisks (*, parental) and pound symbol (#, *Klf1*3-KO) indicate statistically significant differences (*p* < 0.05) when comparing the mean of treatments with the control within each cell line. Points represent the mean ± SEM (*n* = 4/treatment). (**E**–**H**). To silence the expression of *Stat3*, we transfected the *Klf13*-KO cell line with the plasmid pGIPZ-*Stat3*-shRNA, using the plasmid pGIPZ-nt-shRNA as a control. Silencing was allowed for 5 days before treating the cells with 1 nM GH for 1 h. The cells were harvested to quantify the expression of the JAK/STAT output genes. GH-induced expression of *Socs1* (**E**) and *Socs3* (**F**) was completely blocked by *Stat3* silencing (GH-nt-shRNA vs. GH-*Stat3*-shRNA: *Socs1*, *p* = 0.0163; and *Socs3*, *p* = 0.0284), while the expression of *Igf1* (**G**) and *Bdnf* (**H**) was significantly reduced, although not to baseline levels (GH-nt-shRNA vs. GH-*Stat3*-shRNA: *Igf1*, *p* = 0.0014; and *Bdnf*, *p* = 0.0074; two-way ANOVA followed by Tukey’s multiple comparison post hoc test). Bars represent the mean ± SEM (*n* = 4/treatment), and letters indicate statistically significant differences (*p* < 0.05). Means with the same letter (a, b or c) are not significantly different.

We performed a GH time-response experiment in both parental and *Klf13*-KO HT22 cells to quantify the mRNA expression of *Socs1*, *Socs3*, *Igf1*, and *Bdnf*. The results showed that mRNA levels of *Socs1* were significantly reduced to 0.36 ± 0.2-fold at 4 and 16 h after GH treatment in HT22 parental cells. However, in *Klf13*-KO cells, the expression of *Socs1* was induced by GH by 1.8 ± 0.1- and 2 ± 0.08-fold, at 1 and 8 h after GH treatment, respectively. It is noteworthy that *Socs1* mRNA levels were 3–6 times higher in KLF13-depleted cells compared to parental cells (Figure 4A). In contrast to the effect on *Socs1*, the expression of *Socs3* was significantly induced by GH in both cell lines, showing a similar pattern, although the magnitude of the response was clearly higher in *Klf13*-KO cells. In the parental line, the *Socs3* mRNA levels showed a peak (9.9 ± 0.07-fold) after 1 h of GH treatment as compared to vehicle-treated cells, returned to basal levels after 4 h, and then increased again after 8 h of treatment (4.2 ± 0.03-fold). A parallel, but stronger effect was also observed in *Klf13*-KO HT22 cells, showing a 7.6 ± 0.1- and 5.1 ± 0.2-fold increase after 1 and 8 h of GH treatment, respectively, compared to *Klf13*-KO vehicle-treated cells (Figure 4B).

The gene expression of *Igf1* in the parental line was significantly induced by GH at 2 h post-treatment (1.7 ± 0.03-fold), peaked at 8 h (2.7 ± 0.07-fold), and held up to 16 h after treatment. In *Klf13*-KO cells, however, the GH-dependent expression of *Igf1* reached a significant 3.8 ± 0.18-fold induction until 8 h post GH treatment, and although its mRNA levels decreased after 16 h, they were still significantly higher (2.5 ± 0.12-fold) than in vehicle-treated cells (Figure 4C. The expression of *Bdnf* was also induced by GH in the parental cell line, with the highest mRNA levels during the first two hours after treatment (up to 1.6 ± 0.06-fold), followed by a return to baseline. In *Klf13*-KO cells, the mRNA expression of *Bdnf* was induced by GH starting at 30 min after treatment (1.6 ± 0.05-fold) and kept at a higher level across the evaluated time points (up to 2.8 ± 0.08-fold), although with an oscillating expression pattern (Figure 4D).

### 2.6. STAT3 Mediates the Enhanced GH-Induced Expression of the JAK/STAT Output Genes in KLF13-Deficient HT22 Cells

Based on our findings, which showed that the mRNA expression and protein levels of STAT3 were higher in KLF13-deficient HT22 cells compared to the parental line, we hypothesized that STAT3 mediates the enhanced expression of JAK/STAT output genes induced by GH in KLF13-depleted cells. Therefore, we performed shRNA-mediated silencing of the *Stat3* mRNA in *Klf13*-KO HT22 cells. Firstly, we validated that transfection of the plasmid pGIPZ-*Stat3*-shRNA into the HT22 parental line reduced the endogenous STAT3 protein content by 67 ± 22% (*p* = 0.036, Student’s *t*-test) in comparison with cells transfected with the control plasmid pGIPZ-nt-shRNA. Then, we analyzed the expression of JAK/STAT output genes induced by GH in *Klf13*-KO HT22 cells and found that the silencing of the *Stat3* mRNA completely blocked the GH-induced expression of *Socs1* and *Socs*3 genes (Figure 4E,F). For both genes, two-way ANOVA analysis revealed statistically significant main effects of GH treatment (*Socs1*: *F*_(1,12)_ = 14.39, *p* = 0.0026; *Socs3*: *F*_(1,12)_ = 5.71, *p* = 0.024) and *Stat3* silencing (*Socs1*: *F*_(1,12)_ = 15.09, *p* = 0.0022; *Socs3*: *F*_(1, 12)_ = 7.43, *p* = 0.018) but not significant interaction effect.

On the other hand, silencing of *Stat3* reduced the GH-mediated induction of *Igf1* expression from 2.2 ± 0.03- to 1.7 ± 0.04-fold, and the two-way ANOVA exhibited significant effects of GH treatment (*F*_(1,12)_ = 207.2, *p* < 0.0001), *Stat3* silencing (*F*_(1,12)_ = 12.59, *p* = 0.004), and significant interaction between factors (*F*_(1,12)_ = 12.90, *p* < 0.0038, Figure 4G). For *Bdnf*, GH treatment increased mRNA levels to a similar extent in both pGIPZ-nt-shRNA- and pGIPZ-*Stat3*-shRNA-transfected cells, with fold changes of 1.17 ± 0.03 and 1.28 ± 0.01, respectively. However, absolute levels of *Bdnf* mRNA were significantly lower in *Stat3*-silenced cells (Figure 4H). Two-way ANOVA showed significant main effects of GH treatment (*F*_(1,12)_ = 30.92, *p* = 0.0001) and *Stat3* silencing (*F*_(1,12)_ = 38.99, *p* < 0.0001) but not significant interaction.

### 2.7. GH Induces Klf13 Expression and KLF13 Synthesis but Not Its Nuclear Translocation

**Figure 5:**
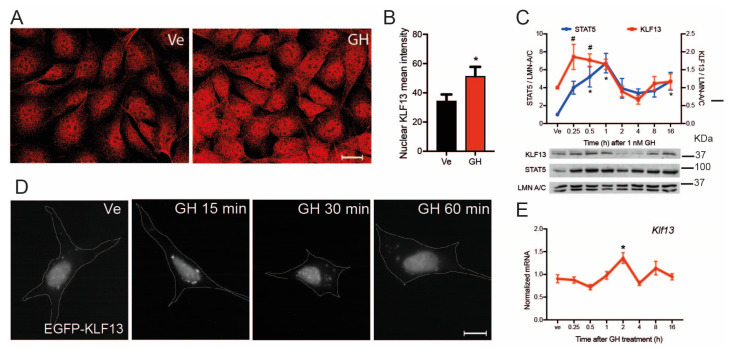
GH induces KLF13 synthesis and *Klf13* expression in HT22 cells. (**A**). The parental HT22 cell line was treated with vehicle or 1 nM GH for 30 min before fixing cells for KLF13 immunocytochemistry using our custom polyclonal anti-mouse KLF13 antibody. Representative images of HT22 cells show that the KLF13 signal increased in cells treated with GH compared to vehicle cells, particularly in the nucleus. (**B**). Quantification of the KLF13 immunofluorescent nuclear signal in cells like those in panel (**A**). Nuclear localization of KLF13 increased significantly in cells treated with GH (*p* < 0.0001; Student’s *t*-test). Bars represent the mean ± SEM (*n* = 25/treatment). (**C**). We then analyzed the nuclear levels of KLF13 after GH treatment, using the STAT5 translocation as a positive control, and laminin A/C as a loading control. HT22 parental cells were treated with 1 nM GH and then harvested at the indicated time points. Nuclear fractions were isolated, proteins extracted, resolved in SDS/PAGE and transferred to nitrocellulose membranes for Western-blot analysis to detect the KLF13 and STAT5 signals. The nuclear levels of KLF13 increased from 0.25 to 0.5 h after GH treatment, while the STAT5 levels were strongly induced by GH at 0.5, 1, and 16 h after treatment (KLF13: *F*_(7,24)_ = 5.2, *p* = 0.001; STAT5: *F*_(7,24)_ = 3.696, *p* = 0.0075; one way ANOVA). Pound symbol (#, KLF13) and asterisks (*, STAT5) indicate statistically significant differences with *p* < 0.05 in Dunnett’s post hoc test comparing the mean of treatments with the vehicle. Points represent the mean ± SEM (*n* = 4/time point). (**D**). The HT22 parental cells were transfected with the plasmid vector pTO-*Egfp-Klf13*, which encodes the chimeric protein EGFP-KLF13, to analyze whether GH treatment induces KLF13 translocation to the nucleus. After 24 h of plasmid transfection, cells were treated with vehicle or 1 nM GH for 15 to 60 min, before being fixed for analysis of the EGFP-KLF13 signal. Representative images show that EGFP-KLF13 is present in the nucleus from the beginning (vehicle), and GH treatment does not increase its nuclear content. (**E**). The effect of GH on the *Klf13* mRNA expression was analyzed in HT22 parental cells. We conducted a time-response experiment by treating cells with 1 nM GH and harvesting them at the indicated time points to quantify the *Klf13* mRNA levels. The levels were significantly higher only after 2 h of GH treatment (*F*_(7,23)_ = 5.004, *p* = 0.0015; one-way ANOVA. Asterisks indicate statistically significant differences with *p* < 0.05 in Dunnett’s post hoc test comparing the mean of treatments with the vehicle). Points represent the mean ± SEM (*n* = 4/time point).

The activated JAK/STAT pathway induces the expression of genes that encode proteins that exert negative feedback in the pathway’s activity, such as the *Socs* family genes. To investigate whether KLF13 is a target of GH-activated JAK/STAT signaling, we evaluated the effects of GH on KLF13 protein synthesis, nuclear translocation, and gene expression. Immunocytochemistry was used to assess KLF13 immunoreactivity in the HT22 parental line, and results showed that GH treatment for 30 min increased the KLF13 immunoreactivity (Figure 5A). Analysis of the signal using a nuclear mask revealed that the mean fluorescence intensity was increased by 52 ± 2% in cells treated with GH, as compared to those treated with vehicle (Figure 5B). We also performed a time course experiment using Western blotting to analyze the effects of GH treatment on the nuclear content of KLF13. The STAT5 nuclear content, which increased up to 6.7 ± 1-fold by GH, was used as a positive control. This experiment confirmed that GH provoked an increase in the nuclear KLF13 signal by 1.8 ± 0.3-fold after 15 min of treatment, and this increase was maintained until 30 min (Figure 5C).

We constructed a plasmid vector that encoded the KLF13 coding sequence fused with the EGFP protein at the N-terminus to investigate whether the GH-induced increase in KLF13 nuclear signal could be due to the promotion of KLF13 translocation. Our results showed that after 24 h of pTO-*Egfp-Kl*f13 transfection, the EGFP signal was located in the nucleus of transfected HT22 cells, and this nuclear signal was not enriched by GH treatment (Figure 5D), suggesting that KLF13 is translocated to the nucleus just after it is synthesized.

We then analyzed whether GH induced *Klf13* expression in HT22 cells and found a significant enrichment of 1.3 ± 0.4-fold after two hours of GH treatment. However, this signal returned to baseline in longer time points of treatment (Figure 5E).

## 3. Discussion

Here, we demonstrate that the GH-induced activation of the JAK/STAT signaling pathway is impacted by the transcription factor KLF13. Our findings indicate that KLF13 regulates the expression of several genes involved in the JAK/STAT pathway and exerts a dual effect on its activity, promoting the STAT5-mediated branch while inhibiting the STAT3-mediated branch. Notably, we observed that depletion of KLF13 in HT22 cells provokes an enhanced response of the pathway output genes to GH stimulation, which is specifically mediated by STAT3. The effect of KLF13 on the JAK/STAT signaling pathway could be part of feedback mechanisms, as suggested by the results showing that GH induces the expression of *Klf13*, further supporting its role in regulating this critical signaling pathway.

In a previous study, where RNA-seq and ChIP-seq analyses were conducted, we discovered that KLF13 has the potential to impact various signaling pathways with crucial roles in the central nervous system, including the JAK/STAT pathway [10]. In this study, we validated that KLF13 functions as a transcriptional repressor of multiple genes, whose protein products constitute the core of the JAK/STAT pathway. We also discovered that in the absence of KLF13, the expression of certain genes, including *Jak1*, *Stat5b*, and *Socs1*, is deregulated, while *Jak2* and *Jak3* gene expression remains similar to that of cells expressing KLF13 (parental line). This apparent inconsistency can be explained by compensation mechanisms that exist between closely related members of the KLF family. It has been reported that KLF9 partially compensates for the lack of KLF13. However, because KLF13 regulates four times more genes than KLF9, this last factor is unable to fully compensate for KLF13′s actions [8,11,36,37].

The regulation exerted by KLF13 on the expression of JAK/STAT pathway genes in HT22 cells is mostly direct and mediated by the binding of KLF13 to the proximal region of target gene promoters. It is well known that KLF13, through its interaction with the Sin3a protein [38], induces the recruitment of the histone deacetylase system, specifically the histone deacetylase protein HDAC-1, to repress gene transcription [39]. Therefore, we hypothesized that the Sin3a-HDAC system is involved in the transcriptional repression of several JAK-STAT-associated genes, including *Jak1*, *Jak2*, *Jak3*, and *Socs1*.

The absence of KLF13 leads to the loss of inhibitory control over *Jak1* and *Socs1*, or stimulatory control over *Stat5b*, resulting in the opposite regulation of these genes, compared to the enforced expression of *Klf13*. However, the expression of other genes, such as *Stat3*, which is not a direct target of KLF13 since its expression was not modified by forced *Klf1*3 expression and its promoter did not show a KLF13-peak in ChIP-seq dataset [10], is up-regulated in the absence of KLF13. One possible explanation is that the reduced levels of the *Stat5* mRNA levels, as a consequence of KLF13 depletion, could be compensated by *Stat3*. In support of these findings, Von Manstein et al. [40] previously reported in cancer cells that deactivation of STAT5 by blocking the c-Src kinase enzyme leads to increased activity of STAT3, which then takes the main role as an effector of the JAK/STAT signaling in promoting cell proliferation. In addition, it has been demonstrated that in the absence of STAT5a/b, the GH signaling is rerouted to STAT1 and STAT3 in hepatocytes of mice, which partially compensates for the metabolic actions of STAT5 [41].

Our results showing that KLF13 enhances the effect of GH on STAT5 activity by inducing *Stat5* expression support the idea that KLF13 works as a positive mediator of the STAT5 branch in the JAK/STAT signaling pathway. Consistent with these findings, previous studies have reported that KLF13 is necessary for STAT5-dependent signaling in keratinocytes, as *Klf13* silencing results in decreased *Stat5* expression and inhibition of STAT5 branch signaling [42]. On the other hand, to our knowledge, this is the first report about the effects of KLF13 on the STAT3 branch signaling, particularly in the effect of KLF13 depletion on the enhanced activity of the STAT3 branch. These findings are consistent with other studies showing that *Klf11* silencing, a factor closely related to KLF13, stimulates the STAT3 activity, as evidenced by increased phosphorylation of STAT3 during an event of hypoxia/reoxygenation in a rat cardiomyoblast H9c2 cell line [43].

The JAK/STAT pathway is a signaling system used by several molecules to transduce information from the extracellular to the intracellular environment [44]. Interleukins, interferons, and hormones are among the various molecules that activate this signaling pathway [14]. Each molecule activates a specific branch of the JAK/STAT pathway. For example, the pathway mediated by STAT3 preferentially transduces signals from the interleukin families IL-6 and IL-10, as well as hormones such as leptin, while the branch mediated by STAT5 preferentially transduces signals activated by the IL-3 family or hormones such as prolactin, erythropoietin, and growth hormone, among others [13]. Our findings showing that KLF13 is a mediator of the STAT5 branch suggest that KLF13 could be an important factor in the physiological actions mediated by the STAT5 branch, including the actions as a neurotrophic factor performed by GH [45,46]. In this regard, our previous studies demonstrated that *Klf13*-deficient hippocampal neurons exhibit increased cell death following glutamate-induced excitotoxicity [10]. Conversely, both endogenous and administrated GH have been shown to promote cell survival in neurons exposed to various types of injury [28,47,48,49]. Therefore, while we acknowledge that multiple signaling pathways may be involved in this response, it is possible that the depletion of KLF13 could affect cell death by disrupting the JAK/STAT pathway. Further research is needed to fully understand the role of KLF13 in the neuroprotective effects of GH against different types of damage.

Despite its role as a mediator of STAT5, our loss-of-function model demonstrated that depletion of KLF13 results in increased expression of JAK/STAT target genes induced by GH and that this effect is mediated by STAT3. These results have important implications for the physiology and pathophysiology of neurons, as well as for therapeutic approaches. Firstly, this suggests that KLF13 acts as a fine-tuning mediator of the JAK/STAT signaling and that its stable and permanent depletion leads to the atypical activity of this pathway. It is well known that aberrant JAK/STAT signaling is associated with tumorigenesis [13]. For instance, glioma and medulloblastoma are linked to alterations in the JAK/STAT pathway, where STAT3 is the main deregulated STAT [50,51,52]. Interestingly, KLF13 has been identified as a proapoptotic factor [53,54] that can inhibit cell proliferation and invasion of glioma stem cells [55,56]. This suggests that the regulation of JAK/STAT activity mediated by KLF13 could be part of the mechanisms that maintain the homeostatic state of this pathway.

On the other hand, STAT3 has been described as an essential factor in promoting axon regeneration [57]. In vertebrates that maintain the ability to elongate and regenerate axons until adulthood, such as fish, the expression of *Stat3* increases after axon injury and contributes to generating a pro-regenerative genetic program [58]. However, in mammals, although the expression of *Stat3* also increases a few hours after injury [59,60], it is not sufficient to promote axon regeneration, partly due to the intrinsic inhibitory program of neurons [61,62]. It has been demonstrated that KLF13 is an intrinsic inhibitory factor for axon regeneration, in part, by regulating the expression of genes involved in the cAMP pathway [7,8]. These findings, together with those of the present study, point to KLF13 as an interesting target to, by its silencing, enhance the GH-mediated JAK/STAT3 activity and thus stimulate axon regeneration of CNS neurons. This proposal involves a robust action of GH since, as our results showed, the expression of *Igf1* and *Bdnf* induced by GH is strongly enhanced in *Klf13*-deficient cells, and these neurotrophic factors have been shown to have a stimulatory effect on axon elongation and regeneration [63].

Taken together, our findings support the notion that KLF13 is a new regulator of the JAK/STAT signaling pathway in hippocampal neurons. This regulation implies that KLF13 could play an essential role as a mediator of the JAK/STAT signaling in both the physiology and pathophysiology of neurons. To strengthen these findings, further experiments need to be conducted in ex vivo or in vivo systems.

## 4. Materials and Methods

### 4.1. HT22 Cell Cultures

The parental and two modified mouse hippocampus-derived HT22 cell line cultures, which retain characteristics of adult hippocampal neurons [64,65], were employed in this work. One consisted of the HT22 cells previously engineered to control the expression of *Klf13* (V5Klf13) transgene under the control of doxycycline [10], and the other was HT22 cells in which the *Klf13* gene was inactivated using CRISPR/Cas9 genome editing [10]. These cell lines were designated as parental lines (control), HT22-TR/TO-*V5Klf13*, and HT22-*Klf13*-KO. In a previous study [10], we validated that the V5KLF13 protein in the HT22-TR/TO-*V5Klf13* cell line increased, in a time-dependent manner, upon doxycycline induction. Similarly, it was previously corroborated, through Western blotting, that the KLF13 signal was depleted in the HT22-*Klf13*-KO cell line. Cells were cultured in high-glucose DMEM (Invitrogen, Waltham, MA, USA) supplemented with 10% fetal bovine serum (Corning, Somerville, MA, USA) and penicillin G (100 U/mL) plus streptomycin (100 µg/mL; Gibco, Grand Island, NY, USA), at 37 °C under an atmosphere of 5% CO_2_. The culture medium for cells expressing V5Klf13 was additionally supplemented with 5 μg/mL blasticidin (Research Products International, Mt Prospect, IL, USA) and 100 µg/mL zeocin (InvivoGen, San Diego, CA, USA).

### 4.2. Plasmids

The pGL-GAS and pGL-STAT5 plasmids used to analyze the GH-induced JAK/STAT activity in the dual luciferase promoter-reporter assays were kindly donated by Dr. Robert J. Denver (University of Michigan, Ann Arbor, MI, USA).

To silence the expression of *Stat3*, we constructed the pGIPZ-*Stat3*-shRNA and pGIPZ-nc-shRNA (non-coding control) plasmid vectors. First, the oligonucleotides sh*Stat3*-F and sh*Stat3*-R or shNc-F and shNc-R (Table 1) were annealed by incubating them in 10 mM Tris, 50 mM NaCl, 1mM EDTA, pH 7.5, at 95 °C for 5 min, followed by 1 min per step in a ramp of 0.5 °C decreasing until reaching 42 °C. Then, the double-stranded DNA fragment was directionally cloned into the pLV-U6-SGIPZ vector (#174847, Addgene, Watertown, MA, USA) at the *ClaI* and *XbaI* sites.

To study the KLF13 translocation, we fused the encoding sequence of the *enhanced green fluorescent protein* (*Egfp*) at the N-terminus of the *Klf13* encoding sequence. The *Klf13* sequence was amplified from the plasmid pTO-*V5Klf13* using the primers listed in Table 1. We then cloned the amplified fragment into the *KpnI*-digested pTO-*Egfp* plasmid vector using the NEBuilder HiFi DNA Assembly mix (New England BioLabs, Ipswich, MA, USA). The construction of all plasmids was confirmed by Sanger sequencing, at the Unidad de Proteogenómica, INB, UNAM.

### 4.3. Experimental Design

To analyze the effect of forced expression of *Klf13* on the expression of JAK/STAT-involved genes, the HT22-TR/TO-*V5Klf13* cells were plated at a density of 5 × 10^5^ in 6-well plates and induced *Klf13* expression with doxycycline (1 μg/mL) at different time points (0.25, 0.5, 1, 2, 4, 8, and 16 h) of doxycycline exposure. Cells were harvested with TRIzol reagent (Invitrogen, Waltham, MA, USA) and proceeded to extract and purify total RNA. Vehicle-treated cells were used as a control.

To analyze the GH-induced JAK/STAT activity, we first examined the nuclear translocation of STAT3 and STAT5 in HT22-*Klf13*-KO cells as compared to the parental cell line. Cells were plated at a density of 5 × 10^5^ in 6-well plates and allowed to stabilize for 24 h before treatment with 1 nM recombinant bovine GH (National Hormone and Peptide Program; Cat #AFP10325C). The GH dose was chosen based on a dose-response experiment, which demonstrated that 1 nM GH elicited approximately 50% of the maximum response. After 1 h of treatment, cells were rinsed with PBS and processed to obtain nuclear extracts.

For the analysis of GH-induced JAK/STAT activity using the luciferase reporter assay, we used the HT22-*Klf13*-KO cells, with the parental line serving as the control. For the HT22-TR/TO-*V5Klf13* line, cells were treated with either vehicle (control) or doxycycline (1 μg/mL). Cells were plated at a density of 2 × 10^4^ in 24/well plates and stabilized for 20 h; later, they were co-transfected with pGL-GAS or pGL-STAT5 plasmids plus pRenilla, using the Lipofectamine 2000 reagent (Invitrogen, Waltham, MA, USA), for additional 20 h. The HT22-*Klf13*-KO and parental cells were treated with vehicle or 1 nM GH, while in the HT22-TR/TO-*V5Klf13* cells, the GH treatment was performed with or without *Klf13* induction (dox-treated cells). After 20 h of GH treatment, a dual-luciferase reporter assay was performed to analyze the JAK/STAT activity.

To further analyze whether KLF13 could impact JAK/STAT activity after its activation by GH, we conducted an experiment using HT22-TR/TO-*V5Klf13* cells. The JAK/STAT activity was induced with GH and then induced *Klf13* expression. Cells were plated, stabilized, and transfected as described above, and then treated with 1 nM GH. Cells were treated with doxycycline (1 μg/mL) at different time points before harvesting (2, 4, 8, and 16 h) so that they were exposed to GH for different durations before Klf13 induction (18, 16, 8, and 4 h, respectively). The control group did not receive any treatment, and the maximum effect of GH was obtained from cells treated with GH for 20 h without *Klf13* inductions. The cells were then harvested to perform the dual-luciferase reporter assay.

In the *Stat3* silencing experiments, HT22-*Klf13*-KO cells were plated at a density of 2.5 × 10^5^ in 6-well plates and allowed to stabilize for 20 h. We then transfected cells with either the pGIPZ-nt-shRNA (control) or pGIPZ-S*tat3*-shRNA plasmid vectors to silence *Stat3* expression for four days. After this period, cells were treated with either vehicle or 1 nM GH for 16 h before harvesting using TRIzol reagent (Invitrogen, Waltham, MA, USA) for analysis of gene expression.

### 4.4. RNA Extraction and RT-qPCR

Total RNA was extracted from HT22 cells using the TRIzol Reagent (Invitrogen, Waltham, MA, USA) following the manufacturer’s instructions. The RNA was then purified using the Direct-zol RNA Mini Prep kit (Zymo Research, Irvine, CA, USA) and treated with DNase on-column following the manufacturer’s instructions. For each sample, cDNA was synthesized using the High Capacity Reverse Transcription Kit with ribonuclease inhibitor (Applied Biosystems, Waltham, MA, USA)) from 1 μg total RNA. We conducted quantitative real-time PCR using a sequence detection system QuantStudio (Applied Biosystems, Waltham, MA, USA) with Maxima SYBR-Green Master Mix reagent (Thermo Fisher Scientific, Waltham, MA, USA). The used oligonucleotide primers were designed to span exon-exon boundaries where possible (Table 1) using the BLAST primer algorithm (https://www.ncbi.nlm.nih.gov/tools/primer-blast/ (accessed on 1 June 2022)). We generated standard curves by pooling cDNA samples and preparing serial dilutions for relative quantification and normalized all genes to the geometric mean of the mRNA levels of the reference genes peptidylprolyl isomerase A (*Ppia*) and glyceraldehyde-3-phosphate dehydrogenase (*Gapdh*), whose mRNAs were unaffected by the treatments.

### 4.5. KLF13-Peaks Visualization

We analyzed data from a previously published study [10] in which chromatin-streptavidin precipitation sequencing (ChSP-seq) was conducted to identify sites across the genome where KLF13 associates with chromatin. We identified the KLF13 peaks associated with proximal promoters of the *Klf16* (positive control) *Jak1*, *Stat5b*, *Socs1*, and *Socs3* genes and visualized them using the Integrative Genome Viewer IGV [66].

### 4.6. Chromatin Extraction and Immunoprecipitation

We extracted chromatin from HT22-TR/TO-*V5Klf13* cells treated with vehicle or doxycycline (1 µg/mL) for 16 h and conducted ChIP assays as described previously [10,11,67]. Briefly, cells were washed with PBS and crosslinked with 1% paraformaldehyde. The paraformaldehyde was quenched with 125 mM glycine before harvesting cells in ChIP lysis buffer (50 mM HEPES-KOH pH 7.5; 140 mM NaCl; 1 mM EDTA pH 8; 1% Triton X-100; 0.1% sodium deoxycholate; 0.1% SDS) supplemented with the Complete protease inhibitors cocktail (Roche, Basel, Switzerland). The cell lysates were sonicated using a GE 130PB sonicator (Cole-Parmer, Vernon Hills, IL, USA) for 15 cycles of 15 s of sonication with 10 amplitude output power. The chromatin shearing (200–600 bp) was confirmed by agarose gel electrophoresis. For each ChIP reaction, 50 µg of chromatin was incubated with 1 µg of our custom affinity-purified anti-KLF13 IgG [10] in 1 mL of dilution buffer (16.7 mM Tris-HCl, pH 8.1; 150 mM NaCl; 0.01% SDS; 1.1% triton X-100; 1.2 mM EDTA), and rocked at 4 °C for 1 h (5% of chromatin was reserved as input). After incubation, 50 μL of Dynabeads Protein A (Invitrogen, Waltham, MA, USA) was added and incubated overnight at 4 °C. Chromatin complexes were washed seven times with ChiP RIPA buffer (50 mM HEPES pH 8; 1 mM EDTA; 1% NP-40; 0.7% sodium deoxycholate; 0.7% LiCl) as described previously [68]. The crosslinks were removed by incubation at 65 °C overnight (from this step the input samples were processed simultaneously with the ChIP samples). The chromatin was then treated with 10 U of Proteinase K (New England BioLabs, Ipswich, MA, USA) at 40 °C for 2 h followed by 2 µg of RNase A (Applied Biosystems) at 37 °C for 1 h. The DNA was extracted with phenol:choloroform:isoamyl alcohol (Invitrogen, Waltham, MA, USA) and precipitated with 0.3 M sodium acetate and 100% ethanol. Subsequently, the DNA was resuspended in 100 µL nuclease-free water and analyzed using targeted qPCR. We conducted relative quantification of the immunoprecipitated DNA by qPCR using the Maxima SYBR-Green Master Mix reagent (Thermo Fisher Scientific, Waltham, MA, USA) with standard curves prepared from serial dilutions of genomic DNA isolated from HT22 cells using the DNeasy Blood & tissue Kit (Qiagen, Germantown, MD, USA). Precipitated samples were normalized as a percentage of the corresponding input sample. The enrichment of KLF13-associated peaks for *Klf16* (positive control) *Jak1*, *Stat5b*, *Socs1*, and *Socs3* was quantified using primers that were designed based on sequences identified in the bioinformatic analysis (Table 1).

### 4.7. Dual Luciferase Promoter-Reporter Assays

We conducted promoter–reporter assays using the HT22-TR/TO-*V5Klf13* and HT22-*Klf13*-KO cell lines to investigate whether KLF13 affected the activity of the JAK/STAT signaling pathway induced by GH in both the STAT3 and STAT5 branches. The previously constructed and validated reporter plasmids, pGL-GAS and pGL-STAT5, were used as sensors for the specific activity of the STAT3 and STAT5 branches, respectively [34]. Cells were co-transfected with 195 ng of the pGL plasmids along with 5 ng of the promoter-less pRenilla vector using Lipofectamine 2000 (Invitrogen, Waltham, MA, USA) in a ratio of 3 µL Lipofectamine per μg DNA. After treatments (as described in the experimental design section), cells were harvested to conduct the Dual Luciferase Reporter Assay (Promega, Madison, WI, USA) according to the manufacturer’s instructions. The firefly luciferase values were normalized to the Renilla luciferase values and presented as relative luciferase activity (RLA).

### 4.8. SDS-PAGE and Western-Blot Analysis

To analyze the protein content, either in total extracts or in the nuclear fraction, we extracted proteins from cell cultures. For total extracts, cells were harvested in RIPA buffer (Abcam) supplemented with the Complete protease inhibitor cocktail (Roche, Basel, Switzerland) and homogenized with a GE 130PB sonicator (Cole-Parmer, Vernon Hills, IL, USA) at a power amplitude of 10 for 30 s. For experiments involving protein analysis at a nuclear fraction, cells were harvested in lysis buffer (10 mM HEPES pH7.9; 10 mM KCI; 0.1 mM EDTA; 0.1 mM EGTA; 1 mM DTT) supplemented with the protease inhibitor cocktail. The cells were allowed to swell for 15 min before adding NP-40 to a final concentration of 0.5%. The nuclei were then collected by centrifugation and lysed in nuclei buffer (20 mM HEPES pH 7.9; 0.4 M NaCl; 1 mM EDTA; 1 mM EGTA; 1 mM DTT) supplemented with protease inhibitors. We separated 50 μg of total extracts or 20 μg of nuclei extracts in 10 % SDS/PAGE slabs and transferred them onto nitrocellulose membranes (Bio-Rad, Hercules, CA, USA) at a constant current of 200 mA for a duration of 1.5 h. The nitrocellulose membranes were then blocked with 5% bovine serum albumin (Sigma) in Tris-Buffered Saline (TBS, 0.05 M Tris; 0.15 M NaCl, pH 7.6) for 1 h to prevent non-specific binding. Later, the membranes were incubated with specific primary antibodies (Table 2) diluted in TTBS (0.1% Tween-20 in 1X TBS) at 4° for 16 h. After washing with TTBS, the membranes were incubated with the corresponding HRP-conjugated secondary antibodies (Table 2). Immunoreactive bands were detected using an ECL blotting detection reagent (GE HealthCare Technologies, Chicago, IL, USA) and visualized on autoradiography film (Fujifilm). The signals for GAPDH and laminin A/C (LMN A/C) were used as loading controls in total cell extracts and nuclear extracts, respectively.

### 4.9. Immunofluorescent Staining

The cell cultures were washed with PBS before fixing them (4% paraformaldehyde; 4% sucrose in 1X PBS) at room temperature for 10 min. After three washes with PBS, the cells were permeabilized with 0.1% Triton and blocked them with 2% BSA in PBS. We then incubated them at 4 °C for 16 h with our custom anti-KLF13 primary antibody (Table 2), diluted in the blocking solution. The excess primary antibody was removed by washing the cells three times with PBS before incubating them with a goat anti-rabbit IgG Alexa-594-conjugated secondary antibody (Invitrogen) for two hours. We washed the cells before mounting them using the Fluoro-gel reagent (Electron Microscopy Sciences, Hatfield, PA, USA). The images were acquired under a red fluorescent filter with an Olympus BX51 fluorescence microscope (Olympus, Tokyo, Japan) using a 20× objective. For each treatment, we observed at least 10 random fields and analyzed a minimum of 30 neurons per treatment from three independent experiments using the Fiji platform [69].

### 4.10. Statistical Analysis

All data are expressed as the mean ± standard error of the mean (SEM). We analyzed data by Student’s independent *t*-test or by one-way analysis of variance (ANOVA) followed by Tukey’s multiple comparison test or by two-way ANOVA followed by Dunett’s multiple comparison test using Prism8 (GraphPad, San Diego, CA, USA). Derived values were Log10-transformed before analysis. A *p* value < 0.05 was considered statistically significant.

## Figures and Tables

**Table 1:** Oligonucleotide sequences for cloning, reverse transcriptase quantitative PCR (RTqPCR), and chromatin immunoprecipitation assays.

To Create the Vectors	Forward (5′–3′)	Reverse (5′–3′)
shStat3	AAAATCGATGGGTGAAATTGACCAGCAATACGAATATTGCTGGTCAATTTCACCCTTTTTTTCTAGAAAA	TTTTCTAGAAAAAAAGGGTGAAATTGACCAGCAATATTCGTATTGCTGGTCAATTTCACCCATCGATTTT
shNc	AAAATCGATGCGCGATAGCGCTAATAATTTCGAAAAATTATTAGCGCTATCGCGCTTTTTTTCTAGAAAA	TTTTCTAGAAAAAAAGCGCGATAGCGCTAATAATTTTTCGAAATTATTAGCGCTATCGCGCATCGATTTT
pTOEgfp-Klf13	TTTAAACTTAAGCTTGGTACCACCATGGCGCCCATGGCAG	TTTAAACTTAAGCTTGGTACCACCATGGCGCCCATGGCAG
**For RT-qPCR**		
Gadph-mRNA	TGTGTCCGTCGTGGATCTGA	CTTCACCACCTTCTTGATGTCACT
Ppia-mRNA	GGTTCCTCCTTTCACAGAAT	AATTTCTCTCCGTAGATGGAC
Jak1-mRNA	CAAGTCTAGTGACCCTGGCA	CAGATTTCCCAGAGCGTGGT
Jak2-mRNA	TTGGGCAAGCTGAAGGAGAG	CATGCCTGGTTGACTCGTCT
Jak3-mRNA	GAACCTGGGTCACGGTTCTT	GCGGGTAGGATACTTGGCTC
Stat3-mRNA	TGGATGCGACCAACATCCTG	CAATGGTATTGCTGCAGGTCG
Stat5a-mRNA	CACTCCTGTACTTGGTTCGTCA	CCAGGTCAAACTCGCCATCT
Stat5b-mRNA	GTACTACACACCGGTCCCCT	ATGCATTTGCAAACTCGGGG
Socs1-mRNA	GATTCTGCGTGCCGCTCTC	CGGGGAGATCGCATTGTCG
Socs3-mRNA	CTACGCATCCAGTGTGAGGG	TGAGTACACAGTCGAAGCGG
Ghr-mRNA	AAGTACAGCGAGTTCAGCGA	GGACTGGGGGTAAAATCAGCA
Igf1-mRNA	TGGATGCTCTTCAGTTCGTG	GTGGGGCACAGTACATCTCC
Bdnf-mRNA	GCTCACACTCCACTGCCCAT	TCCCTGACCCATGCCAGAAGA
**For ChIP-qPCR**		
Klf16-Intron	ACTAAACTCCACCCCACAAC	TCTTTCAAACACTCCCTCGC
Klf16-promoter	GTACGCACTACCCTCACCAG	GGTGGGCGTAACTCTCAAAG
Jak1-promoter	GAGCTGACCAGGGGTGAAC	TCCGCCGACATCCTGTTTAT
Stat5b-promoter	TTCTAGACAGCAGGAGCACG	TTCTAGACAGCAGGAGCACG
Socs1-promoter	AGCTCGAAAAGGCAGTCGAA	AGCTCGAAAAGGCAGTCGAA
Socs3-promoter	GGCAGTTCCAGGAATCGGG	GGCAGTTCCAGGAATCGGG

**Table 2:** Antibodies used in Western-blotting (WB) and immunohistochemistry (IHC) assays.

Target	Host/Type	Dilution	Assay	Source	Cat No.
GAPDH	Rabbit/polyclonal	1:2000	WB	Cell Signaling	14C10
LMN A/C	Goat/polyclonal	1:2000	WB	Santa Cruz Biotechnology	SC-6215
Total STAT3	Rabbit/polyclonal	1:1000	WB	Cell Signaling	12640S
Total STAT5	Rabbit/polyclonal	1:1000	WB	Cell Signaling	94105S
KLF13	Rabbit/polyclonal	1:2000	WB	Custom	Avila-Mendoza et al., 2020 [10]
Rabbit IgG	Goat/HRP-conjugated	1:5000	WB	Invitrogen	65–6120
Goat IgG	Rabbit/HRP-conjugated	1:5000	WB	Thermo Fisher Scientific	81–1620
KLF13	Rabbit/polyclonal	1:1000	IHC	Custom	Avila-Mendoza et al., 2020 [10]
Rabbit IgG	Goat/Alexa 594-conjugated	1:1000	IHC	Invitrogen	A-11012

## Data Availability

The data that support the findings of this study are available from the corresponding author upon reasonable request.
